# Was ist neu … in der Hämophiliebehandlung?

**DOI:** 10.1007/s00101-025-01554-1

**Published:** 2025-06-20

**Authors:** Diana Vajcs, Veronika Schmette, Patrick Möhnle

**Affiliations:** 1 Abteilung für Transfusionsmedizin, Zelltherapeutika und Hämostaseologie, LMU Klinikum München, Marchioninistr. 15, 81377 München, Deutschland; 2 Klinik für Anaesthesiologie, LMU Klinikum München, München, Deutschland

## Einführung

Die Hämophilie ist eine seltene schwere angeborene Blutgerinnungsstörung, bedingt durch Fehlen oder reduzierte Aktivität von Faktor VIII (Hämophilie A) bzw. Faktor IX (Hämophilie B). Ohne spezifische Behandlung sind schwere spontane Blutungsereignisse charakteristisch. Der Standard in der Behandlung ist die regelmäßige prophylaktische i.v.-Substitution des fehlenden Gerinnungsfaktors, meist in Form einer Heimselbstbehandlung. Durch die regelmäßige Substitution kann Blutungskomplikationen und Gelenkfolgeschäden vorgebeugt werden [1].

## Das ist neu!

Neben den lange etablierten Faktorkonzentraten ist mit dem Präparat Emicizumab (Hemlibra®) seit 2018 das erste „Non-Faktor“-Präparat in der Blutungsprophylaxe bei angeborener Hämophilie A, sowohl für Patienten mit als auch ohne Antikörper gegen Faktor-VIII (sog. „Hemmkörper“), verfügbar [2, 3]. Emicizumab imitiert als therapeutischer Antikörper die Funktion des Faktor-VIII-Proteins in der Gerinnungskaskade durch Bindung an Faktor IXa und Faktor X. Es wird ein Effekt auf die Blutgerinnung erzielt; dieser entspricht am ehesten einer Faktor-VIII-Aktivität im Bereich von ca. 10–40 % [4]. Dies reicht in der Regel aus, um Spontanblutungen und Blutungen bei Bagatellverletzungen im Alltag zu vermeiden. Vorteile des Präparates sind die subkutane Anwendung, adäquate Wirksamkeit auch bei Vorliegen von Hemmkörpern gegen Faktor VIII sowie die lange Halbwertzeit mit Dosisschemata einmal wöchentlich, einmal alle 2 Wochen oder einmal alle 4 Wochen.

Bei Blutungskomplikationen, Traumata und auch perioperativ bei größeren Eingriffen reicht die hämostatische Aktivität von Emicizumab nicht aus. In solchen Situationen wird zusätzlich Faktor-VIII-Konzentrat (bzw. rekombinanter aktivierter Faktor VIIa bei Hämophilie A mit Hemmkörpern) benötigt, in Dosierungen bzw. Dosisschemata, welche in der Regel der Substitution ohne Emicizumab entsprechen.

Ein Problem stellen in solchen Situationen die laborchemische Messung und Überwachung dar: Unter Emicizumab ist die Messung der aPTT sowie der Faktor-VIII-Aktivität mit konventionellen („Clotting“-)Verfahren nicht sinnvoll möglich, die aPTT wird unter Emicizumab falsch-verkürzt gemessen, die Messung der Faktor-VIII-Aktivität mit üblichen „Clotting-Tests“ bzw. aPTT-basierten Verfahren ergibt unzuverlässige, falsch-hohe Werte. Für die korrekte Messung der Faktor-VIII-Aktivität unter Emicizumab sind spezielle Laborverfahren (chromogener Test) notwendig, welche jedoch nicht flächendeckend vorgehalten werden [5].

Dies ist v. a. unter Notfallbedingungen, z. B. bei akutem Trauma oder dringlichen Operationen, von Bedeutung, insbesondere in Krankenhäusern, in denen spezialisierte Testverfahren nicht vorgehalten werden, und kann Diagnostik und Therapieentscheidungen wesentlich beeinflussen. Wichtig ist somit die frühe Kontaktaufnahme zum behandelnden Hämophiliezentrum des Patienten, um weitere Entscheidungen wie die Gabe von Faktorkonzentraten, Organisation der laborchemischen Untersuchungen oder auch ggf. Verlegung in ein Zentrum abzustimmen.

## Fazit für die Praxis

Was ist bei Blutungsnotfällen, Traumata und Operationen unter einer Therapie mit Emicizumab zu beachten?

- In den nächsten Jahren werden in der Hämophiliebehandlung neben Emicizumab zunehmend weitere neue „Non-Faktor“-Präparate zur Anwendung kommen (z. B. „Rebalancing“ durch Anti-TFPI-Wirkung, Präparate: Concizumab und Marstacimab, sowie Gentherapie).
- Bei Patienten mit Hämophilie und insbesondere bei Anwendung neuer Therapieverfahren gilt: Bei Blutungsnotfällen sowie dringlichen Operationen sollten frühestmöglich eine Kontaktaufnahme mit dem Hämophiliezentrum und die initiale Therapie gemäß den Angaben des Notfallausweises des Patienten erfolgen.
